# Strigolactone GR24 upregulates target genes of the cytoprotective transcription factor Nrf2 in skeletal muscle

**DOI:** 10.12688/f1000research.16172.2

**Published:** 2018-11-14

**Authors:** Shalem Raju Modi, Tarja Kokkola

**Affiliations:** 1 Department of Internal Medicine, Institute of Clinical Medicine, University of Eastern Finland, Kuopio, 70210, Finland

**Keywords:** Strigolactone, GR24, Nrf2, Oxidative stress, Microarray

## Abstract

GR24 is a synthetic strigolactone analog, demonstrated to regulate the development of plants and arbuscular mycorrhizal fungi. GR24 possesses anti-cancer and anti-apoptotic properties, enhances insulin sensitivity and mitochondrial biogenesis in skeletal myotubes, inhibits adipogenesis, decreases inflammation in adipocytes and macrophages and downregulates the expression of hepatic gluconeogenic enzymes. Transcription factor Nrf2 (Nuclear factor (erythroid-derived 2)-like 2) is a master regulator of antioxidant response, regulating a multitude of genes involved in cellular stress responses and anti-inflammatory pathways, thus maintaining cellular redox homeostasis. Nrf2 activation reduces the deleterious effects of mitochondrial toxins and has multiple roles in promoting mitochondrial function and dynamics. We studied the role of GR24 on gene expression in rat L6 skeletal muscle cells which were differentiated into myotubes. The myotubes were treated with GR24 and analyzed by microarray gene expression profiling. GR24 upregulated the cytoprotective transcription factor Nrf2 and its target genes, activating antioxidant defences, suggesting that GR24 may protect skeletal muscle from the toxic effects of oxidative stress.

## Introduction

Strigolactones are carotenoid-derived phytohormones with endogenous roles in regulating plant growth and exogenous roles in establishing symbiosis of host plant with arbuscular mycorrhizal fungi ^1^. Strigolactones induce beneficial effects in mycorrhiza, such as mitochondrial biogenesis and ATP production ^2– 4^. The anti-cancer properties ^5– 8^ and anti-inflammatory potential ^9^ of strigolactones have recently been investigated in mammalian cells. The positive effects of strigolactone analog GR24 in enhancing insulin sensitivity, mitochondrial function and inhibiting adipogenesis and inflammation in insulin-sensitive cells have also been demonstrated ^10, 11^.

Nrf2 (Nuclear factor (erythroid-derived 2)-like 2) activates gene transcription by binding to the antioxidant response element (ARE) in the promoters of its target genes. It regulates multiple biological functions ranging from cellular redox metabolism, detoxification, heme, lipid and glucose metabolism, NADPH generation, autophagy, apoptosis, xenobiotic stress reponse to inflammation by interacting with its target genes, regulating an extensive antioxidant protein network. Nrf2 is associated with disease pathologies like cancer ^12^, hepatotoxicity ^13^, cardiovascular disease ^14^ and neurodegenerative diseases ^15^.

Mitochondria are the major sites of reactive oxygen species (ROS) production and also the targets of their toxic effects. Mitochondrial dysfunction has been associated with the development of insulin resistance, and diabetes is known to induce oxidative stress through the overproduction of ROS and ROS-induced DNA damage ^16^. Nrf2 activation defends against mitochondrial toxins and ROS and affects mitochondrial function by regulating mitochondrial biogenesis, mitochondrial fatty acid oxidation, respiration, ATP production and mitochondrial dynamics ^17^. With its versatile protective mechanism against oxidative stress, pancreatic β-cell apoptosis and insulin resistance, Nrf2 has become a promising therapeutic target for the treatment of type 2 diabetes ^18^.

We have demonstrated that GR24 ameliorates insulin sensitivity, stimulates mitochondrial biogenesis and ATP production and upregulates genes regulating mitochondrial function in L6 myotubes ^10^. Very recently, the efficacy of GR24 in promoting cytoprotective responses via Nrf2 activation was reported in hepatic and macrophage cell lines ^19^. This work reports a transcriptomic study revealing the potential beneficial effects of GR24 in upregulating Nrf2 and its target genes involved in detoxification, carbohydrate and lipid metabolism, heme metabolism, NADPH regeneration and oxidative stress in L6 myotubes, thus contributing to metabolic homeostasis.

## Methods

Results from the methods described in this study have been published previously ^10^, although the analyses described here are published for the first time.

### Cell culture and differentiation

Rat L6 myoblasts (ATCC, Manassas, VA, USA) were maintained in Dulbecco’s modified Eagle’s medium (DMEM 4.5 g/l glucose, Lonza, Basel, Switzerland) supplemented with 10% FBS (Hyclone, Pasching, Austria), 2 mM L-glutamine (Lonza) and 1% penicillin/streptomycin (Lonza) at +37°C in a humidified atmosphere of 5% CO _2_. The cells were seeded in multiwell plates at 2 × 10 ^4^ cells/cm ^2^ one day before starting the differentiation. Myoblasts were differentiated into myotubes by switching into α-MEM media (Gibco, Paisley, UK) supplemented with 2% horse serum (Gibco) and 1% penicillin/streptomycin. GR24 (3E,3aR,8bS)-3-[[(2 S)-4-methyl-5-oxo-2H-furan-2-yl] oxymethylidene]-4,8b-dihydro-3aH-indeno[1,2-b]furan-2-one, Chiralix, Nijmegen, Netherlands) was dissolved in DMSO. The control samples had equivalent DMSO concentration.

### Microarray sample preparation

L6 myotubes were treated with 60 µM GR24 at 7 days of differentiation for 24 h in three independent experiments, resulting in three replicate microarrays in each treatment group. Total RNAs were extracted with RNeasy Mini kit (Qiagen, Hilden, Germany) and RNA quality was assessed with the Agilent Bioanalyzer 2100 (Agilent Technologies, Espoo, Finland). Total RNA (200 ng) was converted to cDNA with Agilent AffinityScript RNase Block, labelled according to manufacturer’s instructions and purified using RNeasy mini spin columns (Qiagen). RNA Spike-In Kit (Exiqon, Vedbaek, Denmark) was used to monitor the success of labelling. Samples were then mixed with blocking agent, fragmentation and hybridization buffer, and were hybridized to Agilent SurePrint G3 Rat GE 8×60 K Microarrays for 17 h at 65°C before washing and scanning with Agilent Scanner G2505C using manufacturer’s protocols. Agilent Feature Extraction software was used to extract data from raw microarray image files ^10^.

### Microarray data processing

The data was processed with limma package (version 3.28.21) of the Bioconductor software ^20^. Microarray data are MIAME compliant. Differentially expressed transcripts were analysed by Bayes moderated t-statistics followed by the Benjamini-Hochberg correction method to control false discovery rate (FDR) with significance threshold set at p < 0.05 ^20, 21^.

## Results and discussion

The top 5 GR24-upregulated genes (glutathione S-transferase alpha 1, metallothionein 1M, heme oxygenase 1, glutathione S-transferase alpha 2, and sequestosome 1) were found to be known targets of Nrf2 ( Table 1). As the prototypical Nrf2 target gene NAD(P)H quinone dehydrogenase 1 ( *Nqo1*) was also found high on the list, it was compelling to look into the extensive list of Nrf2 target genes. We found 56 known Nrf2 target genes ^22^, including Nrf2 itself, to be upregulated by GR24 treatment ( Table 1). Prior to our study, the effects of strigolactones treatment on the mammalian cell transcriptome have only been investigated in human osteosarcoma cells, where strigolactone analogs mainly upregulated the heat shock stress proteins and downregulated the cell cycle ^5^.

**Table 1.** Selected upregulated Nrf2 target genes in L6 myotubes after 24 h treatment with 60 µM GR24.

Gene symbol	Description	Accession	logFC	Fold change	Adjusted P value
Detoxication: Phase I drug oxidation, reduction and hydrolysis					
*Adh7*	Alcohol dehydrogenase 7	NM_134329	1.604	3.04	1.746E-09
*Akr1b1*	Aldo-keto reductase family 1 member B1	NM_012498	1.831	3.56	7.986E-09
*Akr1b8*	Aldo-keto reductase family 1. member B8	NM_173136	2.503	5.67	1.254E-10
*Akr1cl*	Aldo-keto reductase family 1. member C-like	NM_001109900	0.685	1.61	7.675E-06
*Cbr1*	Carbonyl reductase 1	NM_019170	1.060	2.08	3.055E-08
*Nqo1*	NAD(P)H quinone dehydrogenase 1	NM_017000	3.144	8.84	1.602E-10
*Ptgr1*	Prostaglandin reductase 1	NM_138863	3.233	9.40	1.196E-09
Detoxication: Phase II drug conjugation					
*Gsta1*	Glutathione S-transferase alpha 1	NM_031509	4.610	24.42	3.458E-11
*Gsta2*	Glutathione S-transferase alpha 2	NM_001010921	3.676	12.78	1.348E-09
*Gstm1*	Glutathione S-transferase mu 1	NM_017014	0.707	1.63	7.461E-06
*Gstp1*	Glutathione S-transferase pi 1	NM_012577	2.498	5.65	3.862E-10
*Mgst1*	Microsomal glutathione S-transferase 1	NM_134349	2.327	5.02	1.638E-09
*Ugt1a2*	UDP glucuronosyltransferase 1 family. polypeptide A2	NM_001039691	0.169	1.12	3.123E-02
Detoxication: Phase III drug transport					
*Abcb6*	ATP binding cassette subfamily B member 6	NM_080582	0.194	1.14	6.514E-03
*Abcc1*	ATP binding cassette subfamily C member 1	NM_022281	1.420	2.68	2.985E-07
*Abcc2*	ATP binding cassette subfamily C member 2	NM_012833	0.653	1.57	2.263E-03
*Abcc4*	ATP binding cassette subfamily C member 4	NM_133411	1.367	2.58	4.359E-07
*Abcc5*	ATP binding cassette subfamily C member 5	NM_053924	0.807	1.75	4.986E-06
Antioxidant systems					
*Cat*	Catalase	NM_012520	2.205	4.61	7.440E-09
*Gclc*	Glutamate-cysteine ligase, catalytic subunit	NM_012815	1.231	2.35	2.616E-07
*Ggt1*	Gamma-glutamyltransferase 1	NM_053840	2.590	6.02	6.774E-09
*Glrx*	Glutaredoxin	NM_022278	0.717	1.64	9.108E-05
*Gls*	Glutaminase	NM_012569	1.782	3.44	2.165E-09
*Gpx4*	Glutathione peroxidase 4	NM_001039849	0.451	1.37	1.766E-05
*Prdx1*	Peroxiredoxin 1	NM_057114	1.361	2.57	1.274E-06
*Prdx6*	Peroxiredoxin 6	NM_053576	1.291	2.45	9.933E-06
*Slc6a9*	Solute carrier family 6 member 9 (glycine transporter)	NM_053818	2.283	4.87	9.548E-10
*Slc7a11*	Solute carrier family 7 member 11 (cystine/glutamate transporter)	NM_001107673	0.870	1.83	5.449E-06
*Sod1*	Superoxide dismutase 1	NM_017050	0.939	1.92	2.031E-06
*Srxn1*	Sulfiredoxin 1	NM_001047858	2.330	5.03	7.817E-10
*Txn1*	Thioredoxin	NM_053800	1.712	3.28	4.093E-09
*Txnrd1*	Thioredoxin reductase 1	NM_031614	2.193	4.57	1.765E-08
Carbohydrate metabolism and NADPH regeneration					
*G6pd*	Glucose-6-phosphate dehydrogenase	NM_017006	2.184	4.54	7.894E-09
*Idh1*	Isocitrate dehydrogenase 1	NM_031510	1.096	2.14	4.304E-08
*Me1*	Malic enzyme 1	NM_012600	0.860	1.81	8.162E-08
*Pgd*	Phosphogluconate dehydrogenase	NM_001305435	1.920	3.79	1.503E-09
*Taldo1*	Transaldolase 1	NM_031811	1.150	2.22	2.143E-07
*Tkt*	Transketolase	NM_022592	0.989	1.99	2.321E-06
*Ugdh*	UDP-glucose 6-dehydrogenase	NM_031325	0.623	1.54	2.451E-05
Lipid metabolism					
*Acot7*	Acyl-coa thioesterase 7	NM_001146061	0.852	1.80	6.273E-07
*Acox1*	Acyl-coa oxidase 1	NM_017340	0.706	1.63	1.897E-05
*Ces1a*	Carboxylesterase 1A	NM_001190375	1.132	2.19	7.021E-05
*Pnpla2*	Patatin-like phospholipase domain-containing 2	NM_001108509	0.424	1.34	7.480E-05
Heme and iron metabolism					
*Blvra*	Biliverdin reductase A	NM_053850	0.890	1.85	1.973E-06
*Blvrb*	Biliverdin reductase B	NM_001106236	1.497	2.82	5.723E-09
*Fech*	Ferrochelatase	NM_001108434	1.431	2.70	5.570E-09
*Fth1*	Ferritin heavy chain 1	NM_012848	0.877	1.84	4.826E-07
*Ftl1*	Ferritin light chain 1	NM_022500	1.017	2.02	3.213E-07
*Hmox1*	Heme oxygenase 1	NM_012580	3.959	15.55	1.136E-10
Transcription factors and associated proteins					
*Ahr*	Aryl hydrocarbon receptor	NM_013149	0.867	1.82	1.519E-06
*Cebpb*	CCAAT/enhancer binding protein beta	NM_001301715	0.959	1.94	1.102E-06
*Mafg*	Mafg protein	NM_022386	0.718	1.64	2.006E-05
*Mt1m*	Metallothionein 1M	NM_001137564	4.009	16.10	7.970E-10
*Nfe2l2*	Nuclear factor, erythroid 2-like 2 (Nrf2)	NM_031789	0.659	1.58	3.116E-05
*Rxra*	Retinoid X receptor alpha	NM_012805	0.788	1.73	1.677E-05
*Sqstm1*	Sequestosome 1	NM_181550	3.576	11.93	3.458E-11
Ubiquitin ligase substrate adaptor					
*Keap1*	Kelch-like ECH-associated protein 1	NM_057152	1.269	2.41	3.765E-08

Activation of Nrf2 signaling is known to have beneficial effects in cancer, metabolic syndrome, obesity, nephropathy, retinopathy, neuropathy, and β-cell protection. Nrf2 regulates the transcription of genes involved in antioxidant, detoxification and metabolic processes ^23^. In our study, GR24 enhanced the expression of the Nrf2-dependent antioxidant genes *Nqo1* and heme oxygenase 1, which are known to block inflammatory pathways ^24^. GR24 has recently been shown to alleviate inflammation and upregulate these two NRF2 target genes in hepatic and macrophage cells ^19^.

Glutathione and associated enzymes form an important antioxidant defence system. Treatment with GR24 was found to increase root glutathione content in plants ^25^, but there are no previous reports on the effects of strigolactones on the glutathione system in mammalian cells. Our results show that GR24 treatment upregulated glutamate-cysteine ligase, the rate-limiting enzyme in glutathione synthesis ( Table 1). Many components of the glutathione system are upregulated by Nrf2, and the elevations in the expression of glutaredoxin, glutathione peroxidase 4 and several glutathione S-transferases in GR24-treated cells were evident in our results ( Table 1).

## Conclusions

GR24 upregulated the cytoprotective transcription factor Nrf2 and its target genes, which are involved in detoxification, antioxidant systems, carbohydrate and lipid metabolism, heme and iron metabolism, NADPH regeneration and regulation of transcription in L6 myotubes. Future research aimed to elucidate the effects of GR24 in the oxidative stress mechanisms in skeletal muscle at the protein level may provide supporting evidence about the therapeutic potential of this compound.

## Data availability

Microarray raw data have been deposited in the NCBI Gene Expression Omnibus (GEO), accession number GSE90833: https://identifiers.org/geo/GSE90833.
