# Supplementary material for: Strigolactone GR24 upregulates target genes of the cytoprotective transcription factor Nrf2 in skeletal muscle
Source: F1000Res. 2018 Nov 14;7:1459. Originally published 2018 Sep 13. [Version 2] doi: 10.12688/f1000research.16172.2 (PMC6347031; doi:10.12688/f1000research.16172.2)
Supplement: Peer review history

# Peer review: Referee response for version 2

**DOI:** 10.5256/f1000research.18592.r40629

**Published:** 2019-01-24

**Authors:** David A. Hood

**Affiliations:** 1 Muscle Health Research Centre, School of Kinesiology and Health Science, York University, Toronto, ON, Canada

I have reviewed the responses and am satisfied with the responses. I have no further comments to add.

I have read this submission. I believe that I have an appropriate level of expertise to confirm that it is of an acceptable scientific standard.

# Peer review: Referee response for version 2

**DOI:** 10.5256/f1000research.18592.r40630

**Published:** 2018-11-14

**Authors:** Tugba Boyunegmez Tumer

**Affiliations:** 1 Department of Molecular Biology and Genetics, Çanakkale Onsekiz Mart University, Çanakkale, Turkey

Authors have carried out satisfactory changes in the revised MS. In this form, it can be acceptable for indexing.

I have read this submission. I believe that I have an appropriate level of expertise to confirm that it is of an acceptable scientific standard.

# Peer review: Referee response for version 1

**DOI:** 10.5256/f1000research.17660.r39708

**Published:** 2018-10-31

**Authors:** Tugba Boyunegmez Tumer

**Affiliations:** 1 Department of Molecular Biology and Genetics, Çanakkale Onsekiz Mart University, Çanakkale, Turkey

In current research note, a microarray study was carried out to analyze the effect of GR24, a synthetic strigolactone analog, on the gene expression profile of rat L6 myotubes. 60 µM of GR24 was used to treat L6 myotubes at 7 ^th^ day of differentiation for 24 h in three independent experiments. Microarray data was processed and differentially expressed transcripts were analyzed. Upregulated genes were partly presented in Table 1. Accordingly, GR24 upregulated the cytoprotective transcription factor Nrf2 and its several target genes involved in antioxidant defense and phase II detoxification processes. The fold values especially for *GSTa1* and *a2*, *NQO1*, *GGt1*, *Hmox1*, *Mtm1m*, *Sqstm1* were considerably and significantly high (above 6 fold). Authors previously showed that GR24 (60 µM) ameliorates insulin sensitivity, stimulates mitochondrial biogenesis and ATP production and upregulates genes regulating mitochondrial function in L6 myotubes (Modi et al., 2017 ^1^).

 Also, in this previous report it was shown that 9838 transcripts were upregulated and 6315 transcripts were downregulated in L6 myotubes as a result of 60 µM GR24 treatment. Up and down regulated transcripts involved in insulin signaling and mitochondrial function in L6 myaotubes have been presented in this previous paper (Modi et al., 2017 ^1^). In the present research note, authors represented up regulated transcripts involved in oxidative stress and cytoprotective signaling when myotubes were treated with 60 µM GR24. Since the work can be classified as original and the results are remarkable, data deserves to be published in F1000Research as research note. However, the data is limited to reach the conclusion suggesting that GR24 may protect skeletal muscle from the toxic effects of oxidative stress and thus contributing to metabolic homeostasis. In the scope of report, there is no any biological assay or disease modelling evaluating the beneficial effects of GR24 on myotubes. Furthermore, gene expression profiling does not always reflect the changes on the protein level. Regarding these facts, title also should not contain the assumption suggesting that GR24 may protect against oxidative stress in skeletal muscle.

 In my opinion, both conclusion and title need revision and if possible it is better to represent additional data and interpretation in terms of down regulated genes in specified signaling pathways. Also could you please add an explanation about why you chose 60 µM concentration?

I have read this submission. I believe that I have an appropriate level of expertise to confirm that it is of an acceptable scientific standard, however I have significant reservations, as outlined above.

## References

[1] ModiSYaluriNKokkolaTLaaksoM: Plant-derived compounds strigolactone GR24 and pinosylvin activate SIRT1 and enhance glucose uptake in rat skeletal muscle cells. *Sci Rep*.2017;7(1) : 10.1038/s41598-017-17840-x 17606 10.1038/s41598-017-17840-x 29242624PMC5730588

# Response

**Published:** 2018-11-07

**Authors:** SHALEM RAJU MODI

**Affiliations:** University of Eastern Finland, Finland

**We thank the referee for kindly evaluating our research note and for the valid comments and suggestions. We agree with the comments and hence, we changed the discussion part and also the title of the manuscript.**

 Also could you please add an explanation about why you chose 60 µM concentration?

**We initially tested GR24 at various concentrations from 20 µM – 100 µM. We observed that at 60 µM concentration, GR24 significantly upregulated SIRT1 protein expression and enhanced glucose uptake in skeletal muscle cells** **(Sci Rep. 2017; 7(1): 17606.)** **. Moreover, longer exposure to 100 µM GR24 had a small but significant cytotoxic effect. Hence, we have chosen 60 µM to be an ideal concentration for all our experiments.**

# Peer review: Referee response for version 1

**DOI:** 10.5256/f1000research.17660.r38815

**Published:** 2018-10-02

**Authors:** David A. Hood

**Affiliations:** 1 Muscle Health Research Centre, School of Kinesiology and Health Science, York University, Toronto, ON, Canada

Interesting investigation of the effect of a plant-based compound to change gene expression in muscle cells, however the study is very limited in scope.

 1.  All reported data show increases in mRNA. Are there no significant decreases that are noteworthy?

 2.  Have any changes in mRNA using microarrays been verified with Q-PCR? This is standard practice;

 3.  Could a few protein blots be included to strengthen the data and take the interpretation to the protein level?

 4.  The title assumes that the mRNA changes lead to protein changes which would impact oxidative stress.The title needs to be re-worded unless actual measures of oxidative stress are performed.  this would be relatively easy with an oxi-blot, or ROS measure in cells.

I have read this submission. I believe that I have an appropriate level of expertise to confirm that it is of an acceptable scientific standard, however I have significant reservations, as outlined above.

# Response

**Published:** 2018-10-19

**Authors:** SHALEM RAJU MODI

**Affiliations:** University of Eastern Finland, Finland

We thank the referee for carefully evaluating our research note. He has correctly identified that the study has its limitations. As we have been engaged in other studies and have not been able to dedicate more time for continuing on this track, we decided to submit our microarray analyses in the current format. This Research Note format facilitates publishing small studies, such as our study with only a single table.

 1. All reported data show increases in mRNA. Are there no significant decreases that are noteworthy?

 In the current short publication, we do not report all significant findings. A total of 16,153 transcripts were differentially regulated (9,838 up- and 6,315 downregulated) to the GR24 treatment. Some of the differentially regulated transcripts have been described in our previous publication (Modi S, Yaluri N, Kokkola T, et al.: Plant-derived compounds strigolactone GR24 and pinosylvin activate SIRT1 and enhance glucose uptake in rat skeletal muscle cells. *Sci Rep.* 2017; 7(1): 17606.). Nevertheless, the most interesting finding from the microarray experiments was the strong upregulation of Nrf2 target transcripts by GR24 treatment.

 2.  Have any changes in mRNA using microarrays been verified with Q-PCR? This is standard practice;

 3.  Could a few protein blots be included to strengthen the data and take the interpretation to the protein level?

 4.  The title assumes that the mRNA changes lead to protein changes which would impact oxidative stress.The title needs to be re-worded unless actual measures of oxidative stress are performed.  this would be relatively easy with an oxi-blot, or ROS measure in cells.

 These are very valid questions. The suggested qPCR analyses, protein blots and measures of oxidative stress would be essential next steps to verify the changes in gene expression. For that reason, we published our preliminary analyses as a Research Note, as additional analyses would have been required in order to produce a complete full-length article. When drafting the title, we avoided making strong claims on the effects on oxidative stress. After reading the suggestions by the referee, we have reconsidered the title, and will submit a new version of the article with a title “Strigolactone GR24 upregulates target genes of the cytoprotective transcription factor Nrf2 in skeletal muscle”.
